# Seek and destroy! Ubiquitin-mediated regulation of lignin biosynthesis in bamboo

**DOI:** 10.1093/plphys/kiae525

**Published:** 2024-10-04

**Authors:** Dyoni M Oliveira

**Affiliations:** Assistant Features Editor, Plant Physiology, American Society of Plant Biologists; Department of Plant Biotechnology and Bioinformatics, Ghent University, 9052 Ghent, Belgium; VIB Center for Plant Systems Biology, 9052 Ghent, Belgium

Bamboo's rapid growth and remarkable strength make it a sustainable and eco-friendly alternative to traditional timber. Its mechanical properties largely depend on lignin, which reinforces supportive tissues and imparts hydrophobicity to xylem cells, facilitating efficient water and nutrient transport through the vessels (Li et al. 2024). As a complex phenolic polymer, lignin cross-links with cellulose and hemicelluloses in the secondary cell walls of vascular plants, playing a central role in plant architecture, nutrient transport, and adaptation to biotic and abiotic stresses (Boerjan et al. 2003; Oliveira et al. 2020).

Lignin biosynthesis starts with the deamination of phenylalanine to cinnamic acid by the enzyme phenylalanine ammonia lyase (PAL), the gateway toward secondary metabolism and lignin biosynthesis (Fig. 1). Lignin is typically described as being composed of the 3 canonical building blocks: *p*-hydroxyphenyl (H), guaiacyl (G), and syringyl (S), which are derived from the oxidative coupling of the monolignols, *p*-coumaryl, coniferyl, and sinapyl alcohol, respectively (Boerjan et al. 2003; Ralph et al. 2004). Beyond these canonical building blocks, over 35 different lignin monomers have been identified as naturally occurring, highlighting the complexity and plasticity of lignification (Vanholme et al. 2019). The biosynthesis of lignin is tightly regulated by multiple molecular layers. Previous studies have demonstrated the importance of transcriptional and translational regulation of lignin biosynthetic genes. Recent studies have suggested a role for post-translational regulation including protein turnover by ubiquitination in the regulation of lignin biosynthesis (Yang et al. 2021).

**Figure 1.**
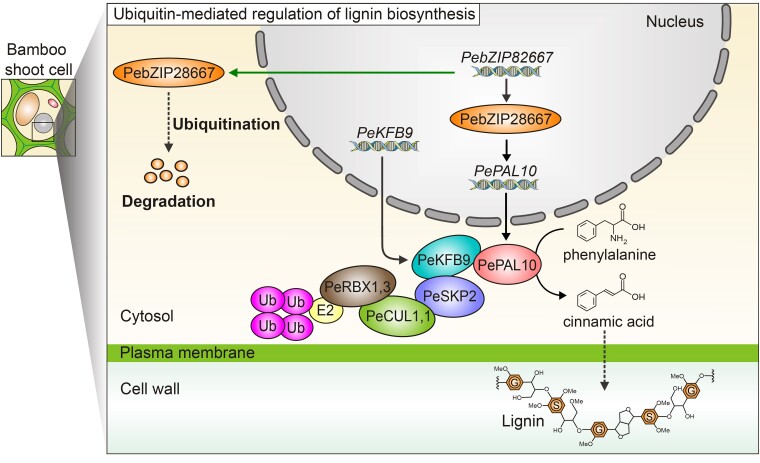
A model of ubiquitin-mediated regulation of lignin biosynthesis in bamboo shoots. PeKFB9 interacts with PeSKP2 and recognizes PePAL10, and the formed complex attaches with the proteins PeSKP2, PeCUL1,1, PeRBX1,3, and E3 ligase to be further ubiquitinated. PePAL10 deaminates phenylalanine to cinnamic acid toward secondary lignin biosynthesis.

In this issue of *Plant Physiology*, Yang et al. (2024) integrated proteomics and ubiquitylomics approaches to investigate the complex posttranslational mechanisms regulating lignin biosynthesis during fast growth of moso bamboo (*Phyllostachys edulis*), a species widely used for building. The authors sampled the 13th internode from bamboo of different heights to examine changes during growth. They observed decreasing protein ubiquitination with increasing shoot maturity, with the highest levels found in the young 2-m shoots sampled and the lowest levels in the older 8-m shoots sampled. They therefore used these 2 tissues for further studies.

The small polypeptide ubiquitin is involved in a wide range of biological processes. It is most characterized for its role in post-translational modification, where it tags proteins for recognition and degradation by the proteasome (Hermand 2006). To generate a comprehensive ubiquitinome dataset for moso bamboo shoots, the authors used label-free ubiquitin enrichment techniques. This approach led to the identification of 13,015 ubiquitination sites across 4,849 proteins in bamboo. Yang and colleagues then turned to identifying lysine ubiquitination (Kub) sites and differentially expressed ubiquitinated proteins. In the later growth stage compared with the earlier stage, the authors found 882 proteins with 2,292 Kub sites that were upregulated, while 1,234 proteins with 2,946 Kub sites were downregulated. A substantial number of ubiquitinated sites were identified in lignin biosynthetic enzymes, including PAL, 4-coumarate:CoA ligase, cinnamyl alcohol dehydrogenase, and cinnamoyl-CoA reductase, highlighting the role of ubiquitination in regulating lignin biosynthesis. Among these, PAL had the highest number of ubiquitinated sites, indicating its significant role in regulating the lignin pathway.

Previous studies have shown that Kelch repeat F-box (KFB) proteins function as a E3 ubiquitin ligases, targeting specific proteins for ubiquitination to regulate various metabolic processes, including phenylpropanoid biosynthesis (Zhang et al. 2013; Li et al. 2024). The authors previously showed that PeKFB9 regulates lignin polymerization by mediating the degradation of peroxidase (Yang et al. 2021). Here, they showed that PeKFB9 specifically interacts with PePAL10, but not with other PePAL proteins.

To further explore the biological function of PeKFB9, the authors overexpressed *PeKFB9* in rice (*Oryza sativa*), which resulted in inhibited growth, reduced biomass, shorter stems, and smaller leaves in the transgenic lines. Notably, *PeKFB9*-overexpressing rice lines had reduced PAL activity, leading to a reduction in cinnamic acid production and consequently decreased lignin content. These findings showed that PeKFB9 promotes the degradation of PAL, disrupting lignin biosynthesis and impacting plant growth.

The authors also identified bZIP transcription factors among the ubiquitin-mediated regulation network. They then confirmed that PebZIP28667 binds directly to the *PePAL10* promoter, inhibiting its transcription. Ubiquitination of PebZIP28667 weakens this inhibition, suggesting a sophisticated interplay between ubiquitination and transcriptional regulation in controlling lignin biosynthesis.

In summary, Yang et al. provide significant insights into the post-translational regulation of lignin biosynthesis in bamboo. The identification of PeKFB9 as a negative regulator highlights the importance of ubiquitination in regulating lignin biosynthesis. By targeting PePAL10 for degradation, PeKFB9 effectively reduces the enzyme's availability, thereby impacting lignin deposition in bamboo shoots. The study also reveals the complexity of the regulatory network by demonstrating how PebZIP28667 modulates PePAL10 expression at both transcriptional and post-translational levels (Fig. 1). This dual-layer regulation is crucial for fine-tuning lignin production, allowing bamboo to adapt to various physiological and environmental conditions. Comparative studies could reveal whether similar ubiquitination-based regulatory mechanisms are conserved across different plants and how they contribute to the diversity of lignin structures and functions. In addition, by manipulating the expression or activity of PeKFB9 or other components of the ubiquitin-mediated regulatory module, researchers could develop bamboo varieties with optimized lignin content for specific uses, such as construction materials or biofuels.

## Data Availability

No new data were generated or analysed in support of this research.
